# Disaster Preparedness and Coping Among Older Adults: Empirical Analysis and Virtual Reality Platform Development

**DOI:** 10.1093/geroni/igab046.1356

**Published:** 2021-12-17

**Authors:** Zhen Cong

## Abstract

This symposium included 4 studies that use national and regional data to examine older adults’ disaster preparedness and coping. The first study examined age differences in preparedness for the continuation of COVID-19 with a sample of 443 residents in Dallas, TX. The findings highlight older adults’ resilience and special needs for different types of support during the pandemic. The second study examined the association of having COVID-19 and intergenerational relationships using the COVID-19 module of the Health and Retirement Study with a sample of 3266 respondents. Using a national sample of 1,467 respondents from the 2017 U.S. National Household Survey, the third study examined age differences relationships among the type of disasters (i.e., disasters with different lead-time), response efficacy, and disaster preparedness. The findings highlighted older adults’ unique vulnerability and resilience in different types of disasters. The fourth study discussed a pilot virtual reality platform under development to assist older adults to develop tailored household emergency preparedness plans and practice those plans with simulated extreme weather conditions and warnings for older adults to practice disaster response and develop relevant knowledge and skills as well as test and revise their emergency preparedness plans. Overall, this symposium emphasizes the uniqueness of older adults’ needs, vulnerability, and resilience to disasters.

